# *Bacillus cereus* cytotoxin K triggers gasdermin D-dependent pyroptosis

**DOI:** 10.1038/s41420-022-01091-5

**Published:** 2022-07-04

**Authors:** Yan Zhao, Li Sun

**Affiliations:** 1 CAS and Shandong Province Key Laboratory of Experimental Marine Biology, Institute of Oceanology, CAS Center for Ocean Mega-Science, Chinese Academy of Sciences, Qingdao, China; 2 Laboratory for Marine Biology and Biotechnology, Pilot National Laboratory for Marine Science and Technology, Qingdao, China; 3 College of Earth and Planetary Sciences, University of Chinese Academy of Sciences, Beijing, China

**Keywords:** Cell death and immune response, Cell death

## Abstract

*Bacillus cereus* is well known as a causative agent of foodborne gastrointestinal diseases and systemic non-gastrointestinal diseases. We have recently identified a pathogenic *B. cereus* (named H2) from a deep-sea cold-seep. H2 possesses the pyroptosis-inducing capacity and contains a number of enterotoxins including cytotoxin K (CytK). In the present work, we examined the cytotoxicity of the CytK of H2 to human macrophages. CytK bound macrophages by interaction with the plasma membrane and caused cellular structure damage. CytK−cell interaction triggered rapid pyroptosis mediated by caspase 1-activated gasdermin D (GSDMD). CytK-induced pyroptosis required NLRP3 inflammasome activation, K^+^ efflux, and intracellular Ca^2+^ accumulation. CytK exhibited apparent binding to several cytomembrane lipids, in particular phosphatidic acid, which proved to be essential to CytK-elicited cell death. Together, these results add new insights into the cytotoxic mechanism of CytK.

## Introduction

*Bacillus cereus* is ubiquitously distributed in various environments, ranging from deep-sea to the terrestrial systems, even in the guts of insects and mammals [1–3]. It is a serious foodborne pathogen that can cause severe gastrointestinal diseases characterized by emesis and diarrhea [4]. An estimated 1.4–12% of food poisoning outbreaks worldwide are caused by *B. cereus* [5]. After *Salmonella* spp., *B. cereus* ranks second among the most common bacterial causes of foodborne illness in mainland China [6]. Emetic syndromes due to *B. cereus* are caused by cereulide, a toxin that is usually produced in food before ingestion [7]. Diarrheal symptoms are usually induced by three enterotoxins that belong to the pore-forming toxin (PFT) family: the tripartite non-hemolytic enterotoxin (Nhe), hemolysin BL (Hbl), and the single cytotoxin K (CytK) [4, 7]. In addition, a growing body of evidence indicates that *B. cereus* is also associated with a number of systemic non-gastrointestinal diseases, including sepsis, endophthalmitis, pneumonia, and endocarditis [8, 9]. However, little is known about the virulence factors involved in non-gastrointestinal infections, and it is unclear whether gastrointestinal infection-associated toxins also play a role in non-gastrointestinal infections. Recent research demonstrated that Hbl and Nhe could activate inflammasome and might cause sepsis in mice [10, 11].

CytK, a member of the PFT family, is a single protein with dermonecrotic, cytotoxic, and hemolytic activity [12, 13]. CytK was initially identified in the bacterial strain NVH 391/98 which caused a severe foodborne outbreak [12]. Soon after, a variant (CytK-2) with an 89% sequence similarity to the first CytK (CytK-1) was identified in another strain, NVH 1230/88 [14]. The strain containing CytK-1 was classified as a new species, *Bacillus cytotoxicus*, which differs from the CytK-2-containing species [15]. CytK-1 and CytK-2 both exhibited hemolytic and cytotoxic activities towards Caco-2 cells and Vero cells and were able to cause permeabilization of planar lipid bilayers [12, 14, 16]. However, the action mechanism of CytK at the cellular level remains to be investigated.

Pyroptosis is a type of inflammatory cell death initiated by perturbation of extracellular or intracellular homeostasis [17]. Pyroptosis is executed by a group of proteins called gasdermin (GSDM), which include GSDMA to F in mammals. Gasdermin D (GSDMD) is a key substrate of caspase (Casp) 1 and Casp4/5/11 [18–20]. The cleavage of GSDMD by Casp1 or Casp4/5/11 removes the auto-inhibitory C terminal domain and allows the N-terminal domain to oligomerize and form pores on the cellular membrane. This causes the extracellular release of interleukin (IL)-1β and IL-18, two cytokines with pro-inflammatory properties. Recently, Casp3 cleavage of GSDME, Casp8 cleavage of GSDMC/D, and granzyme A/B cleavage of GSDMB/E have also been found to trigger pyroptosis [21–27].

NLRP3 is an intracellular sensor that detects a variety of stimuli, including bacterial toxins, leading to the formation and activation of the NLRP3 inflammasome [28]. NLRP3 inflammasome activation often leads to GSDMD-mediated pyroptosis [28]. The upstream signals of NLRP3 activation include K^+^ efflux, intracellular Ca^2+^ flux, and alteration in the structure of intracellular organelles [29]. The most recent research showed that the *B. cereus* enterotoxins Nhe and Hbl work together to induce NLRP3 activation and subsequent pyroptosis [10, 11]. However, it is unclear whether NLRP3 and pyroptosis play a role in the cytotoxicity of CytK.

H2 is a pathogenic *B. cereus* strain isolated from a deep-sea cold-seep that can induce pyroptosis by activation of the NLRP3 inflammasome [30]. H2 harbors several cytotoxins, including CytK, but their functions are largely unknown. The present study explored the cytotoxic activity of the CytK from H2. We found that CytK induced pyroptotic cell death mediated by GSDMD. With this observation, we further investigated the mechanism behind CytK-induced cell death. The results of this study provide a new understanding of the pathogenesis of *B. cereus* and the working mechanism of CytK.

## Results

### CytK induces rapid death of dTHP-1 cells

**Fig. 1:**
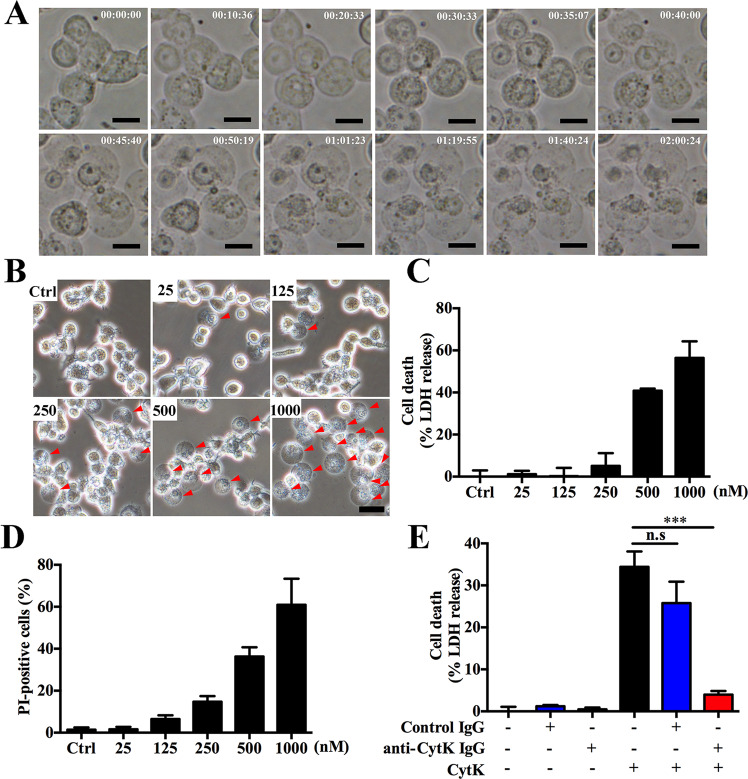
CytK induces rapid death of dTHP-1 cells. **A** Representative time-lapse images of dTHP-1 cells treated with CytK (500 nM). Time: hh:mm:ss. Scale bar, 10 μm. **B** dTHP-1 cells were incubated with or without (control, Ctrl) CytK (25, 125, 250, 500, or 1000 nM) for 1 h and observed with light microscopy. Arrowheads indicate representative cells with a round shape. Scale bar, 10 μm. **C**, **D** dTHP-1 cells were incubated with or without (Ctrl) CytK as (**B**) and assayed for LDH release (**C**) and PI uptake (**D**). **E** dTHP-1 cells were incubated with or without CytK, control IgG, or anti-CytK IgG, and cell death represented by LDH release was measured. For **C**–**E**, data were the means of triplicate experiments and shown as means ± SD. ***p < 0.001, one-way ANOVA with Dunnett’s multiple-comparison test; n.s no significance.

To assess the potential cytotoxicity of CytK, dTHP-1 cells were treated with recombinant CytK at different concentrations. The cells became round, followed by lytic death that was dependent on time and the dose of CytK (Fig. 1A, B and Movie S1). Consistently, CytK dose-dependent increases in LDH release and PI uptake by the cells were observed (Fig. 1C, D and S2). Furthermore, the presence of CytK neutralizing antibodies abolished the cell death-inducing ability of CytK (Fig. 1E). Altogether, these findings indicate that CytK is highly toxic to dTHP-1 cells.

### CytK induces GSDMD-dependent pyroptosis

**Fig. 2:**
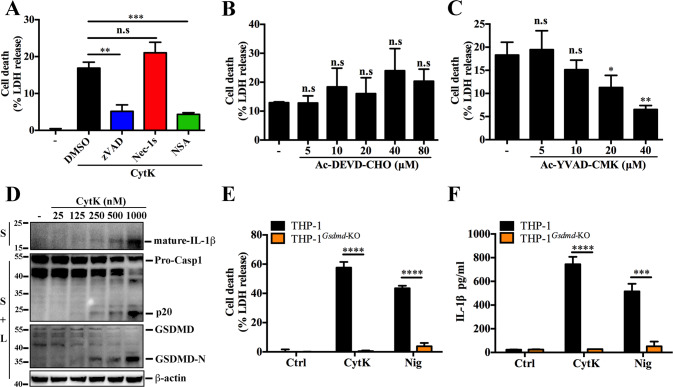
CytK mediates GSDMD-dependent pyroptosis. **A** dTHP-1 cells were pretreated with DMSO, zVAD, Nec-1s, or NSA for 1 h, and then treated with CytK for 1 h. Cell death represented by LDH release was determined. The negative control were untreated cells (−). **B**, **C** dTHP-1 cells were pretreated with or without (−) different concentrations of Casp3 inhibitor Ac-DEVD-CHO (**B**) or Casp1 inhibitor Ac-YVAD-CMK (**C**) for 1 h, and then treated with CytK as above. Cell death represented by LDH release was determined. **D** dTHP-1 cells were treated with or without (−) CytK at increasing concentrations for 1 h. The culture supernatant were mixed with the cell lysate (S + L) and subjected to immunoblotting with antibodies against Casp1, GSDMD, or β-actin (loading control). The culture supernatant (S) alone was immunoblotted with anti-IL-1β antibody. **E**, **F** PMA-differentiated THP-1 and THP-1^*Gsdmd*-KO^ cells were treated with or without (control, Ctrl) CytK (500 nM) or nigericin (Nig, 20 μM) for 1 h, and LDH (**E**) and IL-1β (**F**) release was determined. For **A**–**C**, and **E**, **F**, data were the means of triplicate assays and shown as means ± SD. n.s. no significance; *p < 0.05; **p < 0.01; ***p < 0.001; ****p < 0.0001; one-way ANOVA with Dunnett’s multiple-comparison test (**A**–**C**) or Student’s *t*-test (**E**, **F**).

To elucidate the mechanism of CytK-induced cell death, this study explored the cytotoxic effect of CytK in the presence of various programmed cell death inhibitors. Cell death caused by CytK was significantly hindered in a dose-dependent manner by the pan-caspase inhibitor zVAD and the GSDMD inhibitor NSA, but not by the RIPK1 inhibitor Nec-1s (Figs. 2A and S3). The Casp1 inhibitor Ac-YVAD-CMK, but not the Casp3 inhibitor Ac-DEVD-CMK, also inhibited the cell death caused by CytK in a dose-dependent manner (Fig. 2B, C). These findings imply a vital role of GSDMD and Casp1 in the cytotoxicity of CytK. Indeed, the p20 of activated Casp1 and the pyroptosis-inducing N-terminal fragment of GSDMD (GSDMD-N) were detected in CytK-treated dTHP-1 cells in a fashion that was dependent on the concentration of CytK (Fig. 2D). In addition, mature IL-1β was released from CytK-treated cells (Fig. 2D). Further study using THP-1^*Gsdmd*-KO^ cells demonstrated that following CytK or nigericin (Nig) treatment, both cell death and IL-1β release induced by CytK, as well as Nig, were almost completely abrogated (Fig. 2E, F). These results indicate that CytK induces Casp1- and GSDMD-dependent pyroptosis.

### NLRP3 is required for the cytotoxicity of CytK

**Fig. 3:**
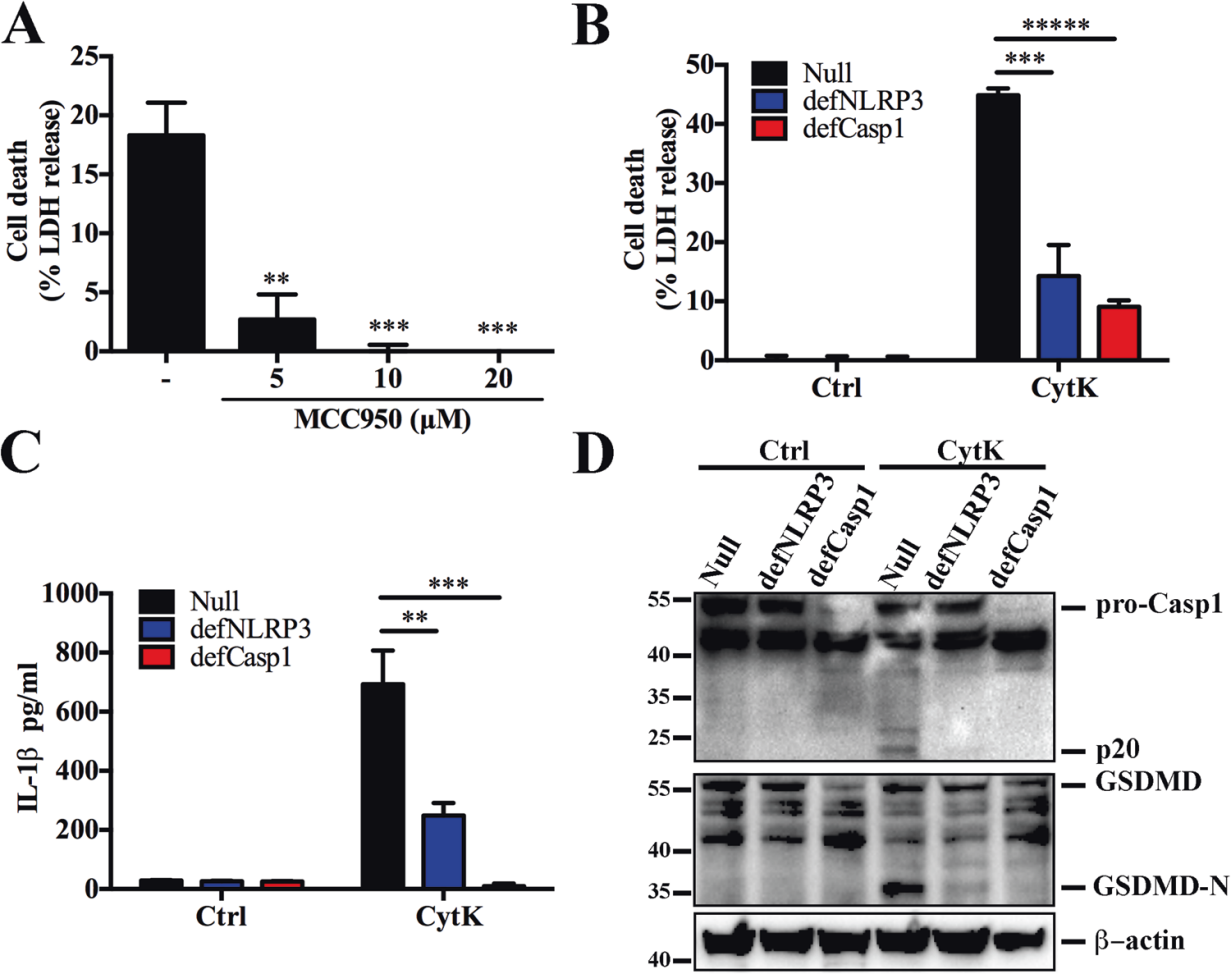
NLRP3 is required for CytK-induced pyroptosis. **A** dTHP-1 cells were pretreated with or without (−) different concentrations of MCC950 for 1 h and then treated with CytK for 1 h. Cell death represented by LDH release was determined. (**B**–**D**) PMA-differentiated THP-1-null, -defCasp1 (*Casp1* knockdown), and -defNLRP3 (*Nlrp3* knockdown) cells were treated with or without (Control, Ctrl) CytK for 1 h. LDH (**B**) and IL-1β (**C**) in the supernatants of the cells were determined. The supernatant together with the corresponding cell lysate were blotted with antibodies against Casp1, GSDMD, or β-actin (loading control) (**D**). For panels **A**–**C**, data were the means of triplicate assays and shown as means ± SD. **p < 0.01; ***p < 0.001; *****p < 0.00001; one-way ANOVA with Dunnett’s multiple-comparison test.

To find out whether NLRP3 was involved in CytK-induced pyroptosis, we examined the cytolytic effect of CytK in the presence of MCC950, an NLRP3-specific inhibitor. MCC950 markedly blocked CytK-triggered cell death at a concentration of as low as 5 μM (Fig. 3A). Consistently, in THP-1-defCasp1 (*Casp1* knockdown) and THP-1-defNLRP3 (*Nlrp3* knockdown) cells, LDH and IL-1β release induced by CytK significantly decreased compared to that in the Null THP-1 cells (Fig. 3B, C). Furthermore, Casp1 activation and GSDMD cleavage were also significantly reduced in defNLRP3 and defCap1 THP-1 cells (Fig. 3D). These results indicate that CytK-triggered pyroptosis depends on NLRP3 and Casp1.

### CytK-induced pyroptosis requires K^+^ efflux and intracellular Ca^2+^ accumulation

**Fig. 4:**
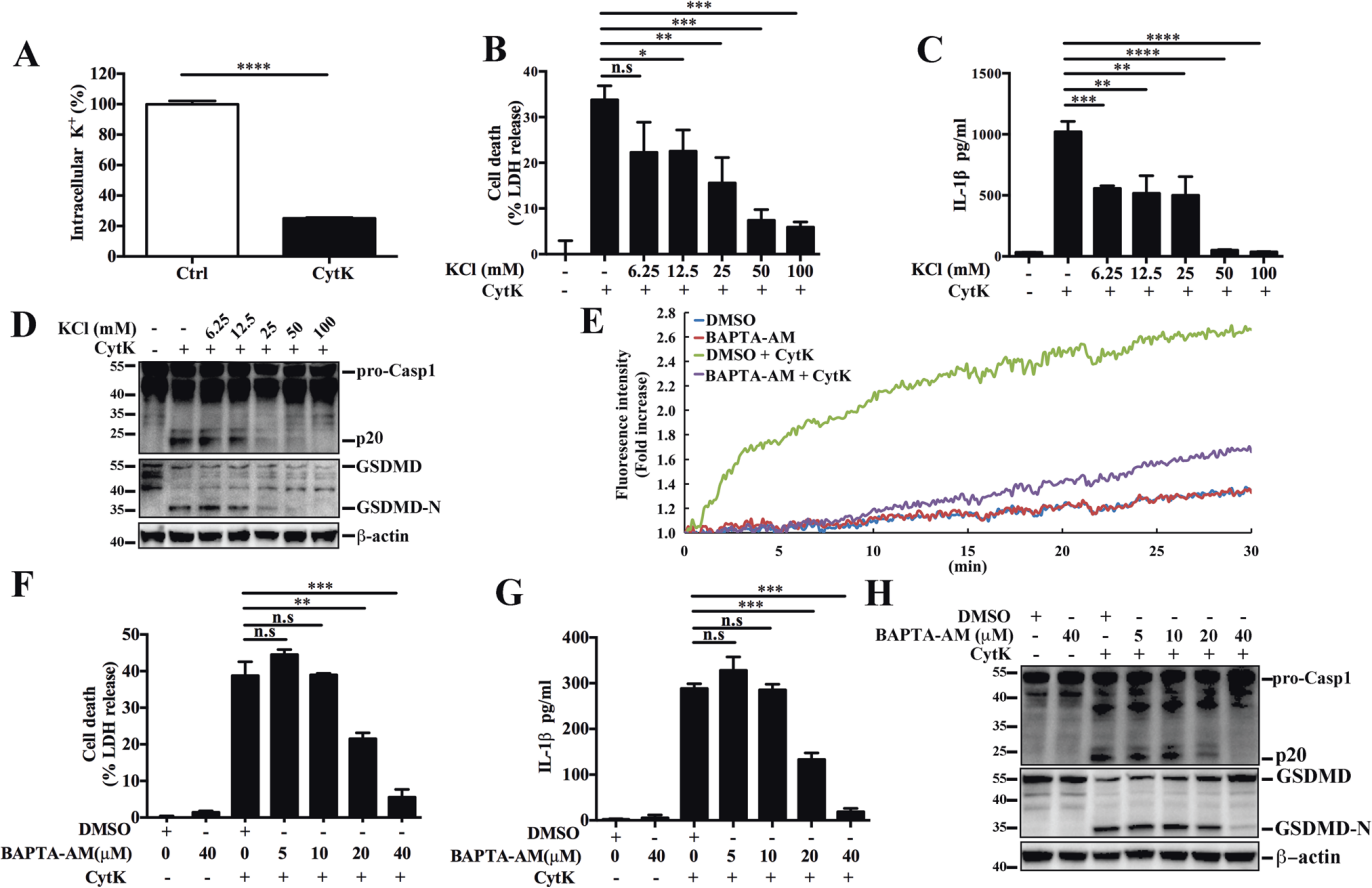
CytK-induced pyroptosis requires K^+^ efflux and intracellular Ca^2+^ accumulation. **A** dTHP-1 cells were incubated with or without (control, Ctrl) CytK for 25 min, and the intracellular concentration of K^+^ was then determined. **B**–**D** dTHP-1 cells were pretreated with or without (−) an increasing dose of KCl for 45 min, and then treated with or without (−) CytK for 1 h. LDH (**B**) and IL-1β (**C**) release in the supernatant was analyzed. The supernatant was also mixed with the cell lysate, and the mixture was immunoblotted to detect Casp1 activation and GSDMD cleavage (**D**). β-actin, a loading control. **E** dTHP-1 cells pre-incubated with Fluo-4 were treated with DMSO or BAPTA-AM for 1 h. The cells were then treated with or without CytK, and the intracellular calcium was measured by determining the fluorescence intensity for 30 min at an interval of 5 s. **F**–**H** dTHP-1 cells were pretreated with DMSO or different concentrations of BAPTA-AM, and then stimulated with or without (−) CytK for 1 h. LDH (**F**) and IL-1β (**G**) release in the cellular supernatant was determined. The supernatant was also mixed with the cell lysate, and the mixture was immunoblotted to detect Casp1 activation and GSDMD cleavage (**H**). β-actin, a loading control. For panels **A**–**C**, and **F**, **G**, data were the means of triplicate assays and shown as means ± SD. *p < 0.05; **p < 0.01; ***p < 0.001; ****p < 0.0001; Student’s *t*-test (A), one-way ANOVA with Dunnett’s multiple-comparisons test (**B**, **C**, **E**, **G**). n.s. no significance.

It is known that potassium efflux triggers the activation of NLRP3 inflammasome [31]. As shown Fig. 4A, we found that the level of intracellular K^+^ significantly decreased in dTHP-1 cells treated with CytK. Supplementation of extracellular K^+^ inhibited CytK-induced cell death, IL-1β release, Casp1 activation, and GSDMD cleavage in a dose-dependent manner (Fig. 4B–D). Besdies K^+^ efflux, intracellular Ca^2+^ also plays a key role in NLRP3 inflammasome-associated pyroptosis [29]. We found that in CytK-treated dTHP-1 cells, the intracellular concentration of Ca^2+^ increased with time (Fig. 4E), which, however, did not occur in the cells pretreated with the intracellular Ca^2+^ chelator BAPTA-AM (Fig. 4E). Further, CytK-elicited cell death and IL-1β release were significantly reduced by BAPTA-AM in a dose-dependent manner (Fig. 4F, G). BAPTA-AM also reduced CytK-induced Casp1 activation and GSDMD cleavage (Fig. 4H). Taken together, these results indicate the importance of K^+^ efflux and intracellular Ca^2+^ accumulation in CytK-induced pyroptosis.

### CytK binds cell membrane and causes membrane damage

**Fig. 5:**
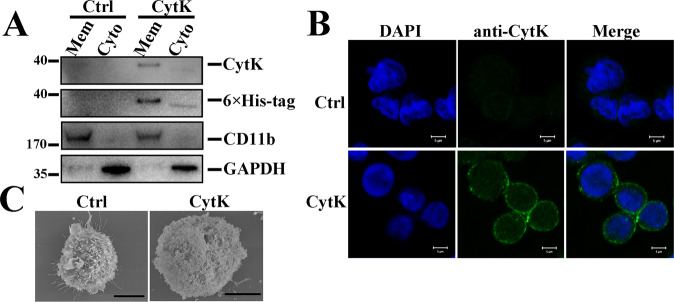
CytK binds the cell membrane and causes membrane damage. **A** dTHP-1 cells were incubated with or without (control, Ctrl) His-tagged CytK for 25 min. The membrane fraction (Mem) and a cytosolic fraction (Cyto) of the cells were immunoblotted with antibodies against CytK, His-tag, CD11b, and GAPDH. CD11b, cell membrane marker; GAPDH, cytosolic protein marker. **B** dTHP-1 cells were incubated with or without (Ctrl) CytK as above and stained with DAPI. The localization of CytK was examined by immunofluorescence microscopy using an anti-CytK antibody. Scale bar, 5 μm. **C** dTHP-1 cells were incubated with or without (Ctrl) CytK for 1 h, and the cells were subjected to scanning electron microscopy. Scale bar, 10 μm.

To examine whether the CytK-induced K^+^ efflux and intracellular Ca^2+^ accumulation observed above were the results of direct interaction between CytK and the cells, we separated the cell membrane and cytosolic fractions of CytK-treated dTHP-1 cells and found that CytK was present in the membrane fraction (Fig. 5A). In line with this observation, immunofluorescence microscopy detected CytK in the plasma membrane (Fig. 5B), and scanning electron microscopy revealed damage to the cellular membrane and structure in CytK-treated cells (Fig. 5C).

### Phosphatidic acid (PA) binds to CytK and plays a crucial role in CytK-induced pyroptosis

**Fig. 6:**
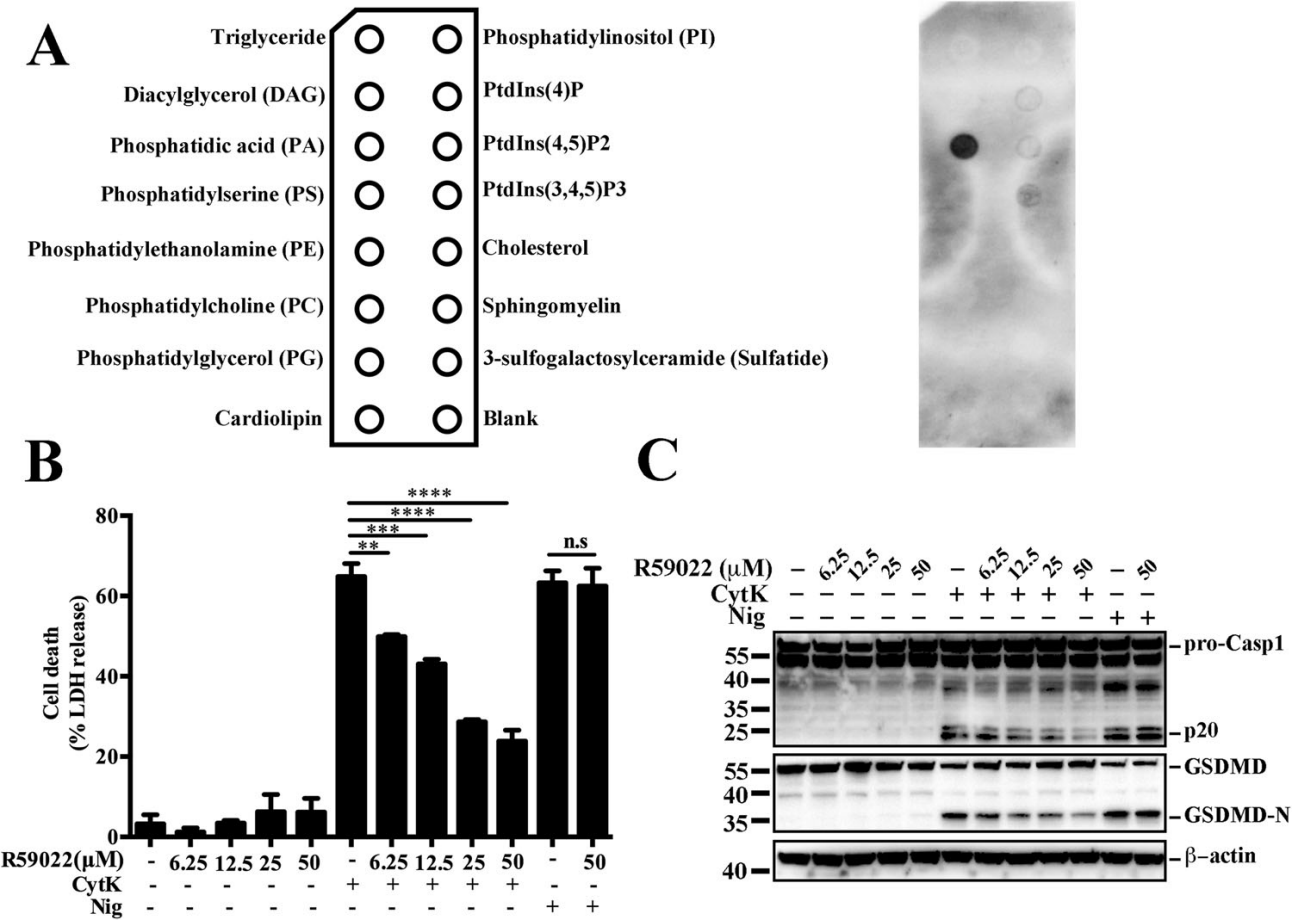
Phosphatidic acid (PA) binds to CytK and is necessary for CytK-induced pyroptosis. **A** A membrane lipid strip pre-coated with indicated lipids (left) were incubated with CytK and then probed with anti-His antibody (right). **B**, **C** dTHP-1 cells were pretreated with or without (−) an increasing dose of R59022 for 6 h, and then treated with or without (−) CytK (500 nM) or nigericin (Nig, 20 μM) for 1 h. LDH release in the cellular supernatants was determined (**B**). The supernatant together with the corresponding cell lysate were blotted with antibodies against Casp1, GSDMD, or β-actin (loading control) (**C**).

Since, as shown above, CytK bound cell membrane, we examined whether CytK could interact with membrane lipids. We found that CytK exhibited apparent binding to phosphatidic acid (PA) and, to lesser extents, PtdIns(4)P, PtdIns(4,5)P2, and PtdIns(3,4,5)P3 (Fig. 6A). The possible role of PA in CytK-induced pyroptosis was then examined using R59022, a small-molecule drug that decreases PA levels [32, 33]. The results showed that CytK-induced cell death was significantly reduced by R59022 in a dose-dependent manner (Fig. 6B). Consistently, Casp1 activation and GSDMD cleavage were also blocked (Fig. 6C). In contrast, R69022 did not affect Nig-induced cell death, Casp1 activation, or GSDMD cleavage (Fig. 6B, C).

## Discussion

*B. cereus* contains several toxins. Among them, the emetic toxin cereulide and the diarrhea-associated enterotoxins Nhe, Hbl, and CytK produce the gastrointestinal and non-gastrointestinal diseases caused by *B. cereus* infection [4, 8, 9, 34]. Nhe, Hbl, and CytK displayed distinct cytotoxic activities on various human cells, suggesting that these toxins may have different modes of action and may act synergistically with each other and other virulence factors to induce disease [4, 7, 8, 16]. Of these toxins, Nhe causes caspase 3-mediated apoptosis in Vero cells [35]. More recently, Nhe, as well as Hbl, have been shown to trigger inflammasome activation and pyroptosis [10, 11]. Compared to Nhe and Hbl, CytK was considered to have the lower cytotoxic potential [16], and the expression of the CytK gene at the mRNA level was not associated with the ability of *B. cereus* to cause cell death in mice macrophages [10, 11]. However, several other studies indicated that *B. cereus* lacking the Hbl and Nhe genes was still pathogenic to humans and able to cause foodborne outbreaks, which may be due to the presence of CytK [7, 12, 15, 36]. In this work, we found that CytK induced cell death at a low concentration, and the cytotoxicity of CytK was dose- and time-dependent, suggesting an important role of CytK in the virulence of *B. cereus*. This finding was consistent with previous research that reported cytopathic effects of CytK [12–14, 16, 36].

Pyroptosis is a form of cell death executed by gasdermins [17–20, 37]. In a previous study, we observed that both the bacterial cells and the culture supernatant of *B. cereus* H2 were able to elicit pyroptosis [30]. A similar observation has been reported by other research groups [10, 11]. In this study, we found that CytK-induced cell death was blocked specifically by pyroptosis inhibitors and required the participation of GSDMD and caspase 1, implying that CytK exerted its cytotoxic effect by activation of GSDMD-mediated pyroptosis. NLRP3 is an upstream activator of pyroptosis. In our study, we found that MCC950, an NLRP3 inhibitor, effectively inhibited Casp1 activation, GSDMD cleavage, and cell death in CytK-treated cells, indicating an important role of the NLRP3 inflammasome in CytK cytotoxicity. Our observations agree with a previous report that administration of MCC950 completely prevented the lethality of *B. cereus* in mice [10]. These observations suggest that the employment of chemical drugs, such as MCC950, targeting NLRP3 might be a promising strategy for the therapy of *B. cereus* infection.

Several stimuli can induce the assembly of the NLRP3 inflammasome complex and initiate pyroptosis [28, 37]. A K^+^ efflux is an upstream event in NLRP3 activation by various NLRP3 agonizts, including Nig, extracellular ATP, bacterial PFTs, and particulate matter [31]. In our study, CytK treatment markedly decreased the intracellular K^+^ concentration, and supplementation of extracellular K^+^ rescued the cells from pyroptotic death. In addition to K^+^, intracellular Ca^2+^ is vital to the NLRP3 complex assembly and activation [29]. Several studies have reported the regulation of NLRP3 activation through a cooperative relationship between K^+^ efflux and Ca^2+^ flux [38, 39]. However, other studies found that K^+^ efflux-mediated NLRP3 inflammasome signaling was not linked to cytosolic Ca^2+^ flux [40, 41]. These findings suggest that the crosstalk between the K^+^ efflux and Ca^2+^ flux pathways in NLRP3 activation is sophisticated and requires further study in order to achieve a more complete understanding [29]. In our study, intracellular Ca^2+^ accumulated after CytK stimulation, and prevention of Ca^2+^ accumulation significantly blocked CytK-induced cell death. Together, these results indicate that CytK caused a change in the status of both K^+^ and Ca^2+^. K^+^ efflux and Ca^2+^ influx may integrate, thereby producing a sufficient signal to activate the NLRP3 inflammasome.

Many pathogenic bacteria produce PFTs, which are important components of the virulence arsenal in these pathogens [42, 43]. PFTs usually disrupt host cell membranes, primarily the plasma membrane, and sometimes intracellular organelle membranes [44]. One report showed that CytK can form pores in planar lipid bilayers [13]. In our study, we found that CytK bound to the cell membrane of THP-1 cells and disrupted the membrane integrity and architecture. Furthermore, CytK exhibited strong binding to membrane PA, which was essential to CytK-induced pyroptosis. PA is a critical plasma membrane component and a precursor for the synthesis of other lipids. In addition, PA plays a key role in mediating diverse cellular and physiological processes in eukaryotes [45]. It is likely that PA binding enabled CytK association and subsequent damage of the plasma membrane that led to K^+^ efflux, which activated NLRP3 and the downstream caspase 1 and GSDMD. Interestingly, a recent study showed PA-mediated binding and cell internalization of the *Vibrio cholerae* cytotoxin MakA [33]. Altogether, these findings indicate that PA plays an important role in the interactions between bacterial toxins and host cells.

In summary, we demonstrated in this study that CytK is a highly effective pyroptosis inducer that disrupts K^+^ and Ca^2+^ homeostasis by damaging the plasma membrane of the target cell via interaction with PA, thus activating the NLRP3−Casp1−GSDMD pathway. These findings reveal new insights into the CytK working mechanism.

## Material and methods

### Cell culture

THP-1 cells were from the Cell Resource Center, IBMS, CAMS/PUMC (Beijing, China). The THP-1 cell line with *Gsdmd* knockout (THP-1^*Gsdmd*-KO^) was generated previously [30]. Cell culture was performed at 37 °C in a 5% CO_2_ humidified incubator. The culture medium was RPMI Medium 1640 basic (1×) (C22400500BT, Gibco) containing 10% (v/v) FBS (10099-141 C, Gibco), 1% penicillin and streptomycin (SV30010, HyClone). THP-1-Null, THP-1-defCasp1 (*Casp1* knockdown), and THP-1-defNLRP3 (*Nlrp3* knockdown) were purchased from InvivoGen and cultured as instructed by the manufacturer.

### Purification of recombinant proteins and preparation of antibodies

The coding sequence of CytK without signal peptide (residues 31–336) was amplified by PCR from the genome of *B. cereus* H2 (GenBank accession number CP043966). The sequence was then cloned into pET-28a at the NcoI/XhoI sites using ClonExpress II One Step Cloning Kit (C112, Vazyme). *E. coli* BL21(DE3) (CD601, TransGen Biotech) was transformed with the recombinant plasmid. The transformed *E. coli* strain was grown in Luria–Bertani (LB) medium at 37 °C until OD_600_ 0.6. Isopropyl-β-d-thiogalactopyranoside (I8070, Solarbio) (0.2 mM) was added to the culture, and the culture was continued at 16 °C for 16–20 h. Bacteria were collected and subjected to sonication in the buffer of 50 mM NaH_2_PO_4_, 300 mM NaCl and 10 mM imidazole. After centrifugation, the pellet was collected and dissolved in the above buffer supplemented with 8 M urea. His-tagged CytK was purified with Ni-NTA Agarose (QIAGEN). Reconstitution of the protein was carried out as reported previously [46]. Rabbit polyclonal antibodies against CytK were generated by Sangon Biotech (Shanghai, China). The purified CytK and its immunoblot with an anti-CytK antibody are shown in Fig. S1.

### Stimulation of THP-1 cells with CytK

To induce differentiation of THP-1 cells (as well as variants) into macrophages (dTHP-1 cells), the cells were pretreated with phorbol 12-myristate 13-acetate (PMA, P1585, Sigma) (50–100 nM) overnight at 37 °C. All stimulation was performed in reduced serum medium Opti-MEM (31985-070, Gibco). The differentiated cells were washed with PBS and incubated with various concentrations of CytK for 1 h. To examine the effects of various inhibitors on CytK activity, the following inhibitors were used to treat dTHP-1 prior to CytK stimulation: Ac-DEVD-CHO (A0835, Sigma), Ac-YVAD-cmk (SML0429, Sigma), Z-VAD-FMK (S7023, Selleck), necrosulfonamide (NSA, S8251, Selleck), necrostatin-1 stable (Nec-1s, S8641, Selleck), BAPTA-AM (S7534, Selleck), R59022 (B6967, ApexBio), or potassium chloride (KCl, 10016318, Sinopharm). To examine the effect of nigericin (serving as a positive control), the cells were treated as above, except that CytK was replaced by 20 μM nigericin (Nig, 481990, Sigma). For the antibody neutralization assay, CytK was pre-incubated with the antibody against CytK (anti-CytK IgG) or the control antibody (rabbit IgG) (D110502, Sangon Biotech) for 1 h before incubation with dTHP-1 cells.

### Immunoblotting

For immunoblotting analysis, dTHP-1 variants (1 × 10^6^ cells/well) in 12-well plates were subjected to the indicated treatment. The cell lysate and culture supernatant used for immunoblot were prepared as described previously [30]. For immunoblotting, the proteins were separated by SDS-PAGE and transferred to nitrocellulose blotting membranes (A29740189, Cytiva). The membranes were blocked in 5% BSA or 5% skimmed milk and then treated with the antibodies targeting the following proteins: Caspase 1 (2225 S, Cell Signaling Technology), GSDMD (96458 S, Cell Signaling Technology), IL-1β (12703 S, Cell Signaling Technology), CD11b (66519-1-Ig, Proteintech), 6×His-tag (ab213204, Abcam), GAPDH (ab181602, Abcam), β-actin (AC004, ABclonal), and CytK. The membranes were then incubated with horseradish peroxidase-conjugated secondary antibody {HRP goat-anti-rabbit (ab97051, Abcam) or HRP-conjugated goat antibody against mouse IgG (AS003, ABclonal)} for 1 h at room temperature. An ECL chemiluminescence kit (P0018S, Beyotime) was used to detect immunoreactive proteins, and imaging was performed with the GelDoc XR System (Bio-Rad).

### Cytotoxicity and IL-1β release analysis

To determine cell death, the CytoTox 96^®^ Non-Radioactive Cytotoxicity Assay kit (G1780, Promega) was used to quantify lactate dehydrogenase (LDH) release from dTHP-1 variants. The release of IL-1β was quantified with a Human IL-1β ELISA kit (EHC002B, NeoBioscience) following the manufacturer’s protocol.

### Microscopy

To examine cell morphology, the cells of dTHP-1 were incubated with 500 nM CytK and then observed with a microscope (Ti-S/L100, Nikon). The movie was processed using Image-Pro Plus 6.0. To examine cell membrane integrity, the cell culture was supplemented with propidium iodide (2 μg/ml) (PI, ST511, Beyotime), and observations were conducted with a confocal microscope (Zeiss LSM 710). The percent of PI-positive cells was calculated at least from five random fields for each experiment. During immunofluorescence microscopy, the cells of dTHP-1 were treated with CytK (500 nM) for 25 min, washed in PBS three times, and then treated with 4% paraformaldehyde for 10 min. After washing as above, the cells were incubated with permeabilization solution (0.2% Triton X-100 in PBS) for 5 min and blocked with 5% BSA for 1 h. The cells were incubated with rabbit anti-CytK (1:100 dilution in 1% BSA) overnight at 4 °C and treated with goat-anti-rabbit antibody (Alexa Fluor 488) (ab150081, Abcam) (1:500 dilution) for 1 h, followed by DAPI (C1002, Beyotime) staining for 10 min. After each incubation step, 0.1% Tween-20 (in PBS) was used to wash the cells. A confocal microscope (Zeiss LSM 710) was used to observe the cells. Scanning electron microscopy was performed as reported previously [47].

### Intracellular K^+^ measurement

For intracellular K^+^ measurement, dTHP-1 cells in 12-well plates (1 × 10^6^ cells/well) were stimulated for 25 min with 500 nM CytK in Opti-MEM medium. Pre-warmed modified phosphate buffer saline (PBS) without K^+^ (NaCl, 140 mM; Na_2_HPO_4_, 10 mM; NaH_2_PO_4_, 2 mM) was used to wash the cells in order to eliminate residual K^+^. The cells were lysed with concentrated nitric acid, and the lysates were analyzed for K^+^ concentration by inductively coupled plasma optical emission spectrometry (ICP-OES) with a PerkinElmer Optima 7300 DV spectrometer.

### Intracellular Ca^2+^ detection

For cytosolic Ca^2+^ detection, dTHP-1 cells in 96-well plates (black plates with clear flat bottom) (3603, Costar) were incubated for 30 min at 37 °C with 4 μM Fluo-4 AM (F14201, Invitrogen) and 0.02% (w/v) Pluronic F-127 (ST501, Beyotime), after which 40 μM BAPTA-AM or DMSO (control) were added. Following incubation for 1 h, CytK (500 nM) was added to the cells, and the cell fluorescence was determined using a Tecan Infinite^®^ M1000 Pro multimode flagship microplate reader with excitation at 494 nm and emission at 516 nm. Measurements at 5-s intervals were used to time-lapse quantify the fluorescence. The obtained fluorescence intensities were normalized to time = 0 to determine the fold increase in intensity.

### Separation of the cellular membrane and cytosolic fractions

The cells of dTHP-1 were incubated with CytK for 25 min, followed by washing in PBS three times. The cells were separated into subcellular fractions by using the Membrane and Cytoplasmic Protein Extraction kit (C510005, Sangon Biotech).

### Protein-lipid binding assay

The binding between CytK and lipids was determined with Membrane Lipid Strips (P-6002, Echelon Biosciences). Briefly, the strips of lipid were pre-incubated with Buffer I {0.1% Tween-20 and 3% fatty acid-free BSA (SRE0098, Sigma) in PBS} for 1 h. The strips were then incubated with CytK (250 nM) in fresh Buffer I for 1.5 h. The strips were washed three times (10 min each time) with Buffer II (0.1% Tween-20 in PBS). The strips were then incubated with horseradish peroxidase-conjugated Mouse anti-His-Tag (AE028, ABclonal) antibody (1:1000) in fresh Buffer I for 1 h. After washing three times (10 min each time) with Buffer II, the lipid-bound CytK was detected with an ECL Western blot detection system described above. The assay was performed at room temperature.

### Statistical analysis

GraphPad Prism 6 (GraphPad Software) was employed for statistical analysis. For two group analysis, student’s *t*-test was used; for three or more group analysis, one-way analysis of variance (ANOVA) was used. Statistical significance was defined as *p < 0.05, **p < 0.01, ***p < 0.001, ****p < 0.0001, and *****p < 0.00001.

## Supplementary information

Supplementary data

The time-lapse images of PMA-differentiated THP-1 (dTHP-1) cells treated with CytK

Original Data File

## Data Availability

All data generated or analyzed during this study are included in the article and its Supplementary Information files.
