# Supplementary material for: Bacillus cereus cytotoxin K triggers gasdermin D-dependent pyroptosis
Source: Cell Death Discov. 2022 Jul 4;8:305. doi: 10.1038/s41420-022-01091-5 (PMC9253000; doi:10.1038/s41420-022-01091-5)
Supplement: Supplementary file 1 — Supplementary data [file 41420_2022_1091_MOESM1_ESM.docx]

**Supplementary data**

**Figure S1 Analysis of purified CytK**. (A) Purified CytK was analyzed by SDS-PAGE and viewed after staining with Coomassie brilliant blue R-250. (B) Purified CytK were immunoblotted with antibodies against CytK and His-tag.

**
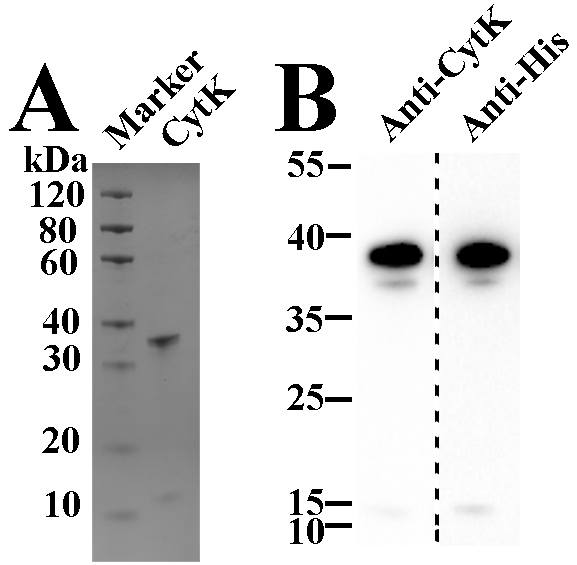
**

**Figure S2** **CytK induces cell death in PMA-differentiated THP-1 (dTHP-1) cells.** Representative images of dTHP-1 cells treated with CytK at different concentrations. The cells were stained with propidium iodide (PI) and observed with a confocal microscope. Scale bar, 10 μm.

**
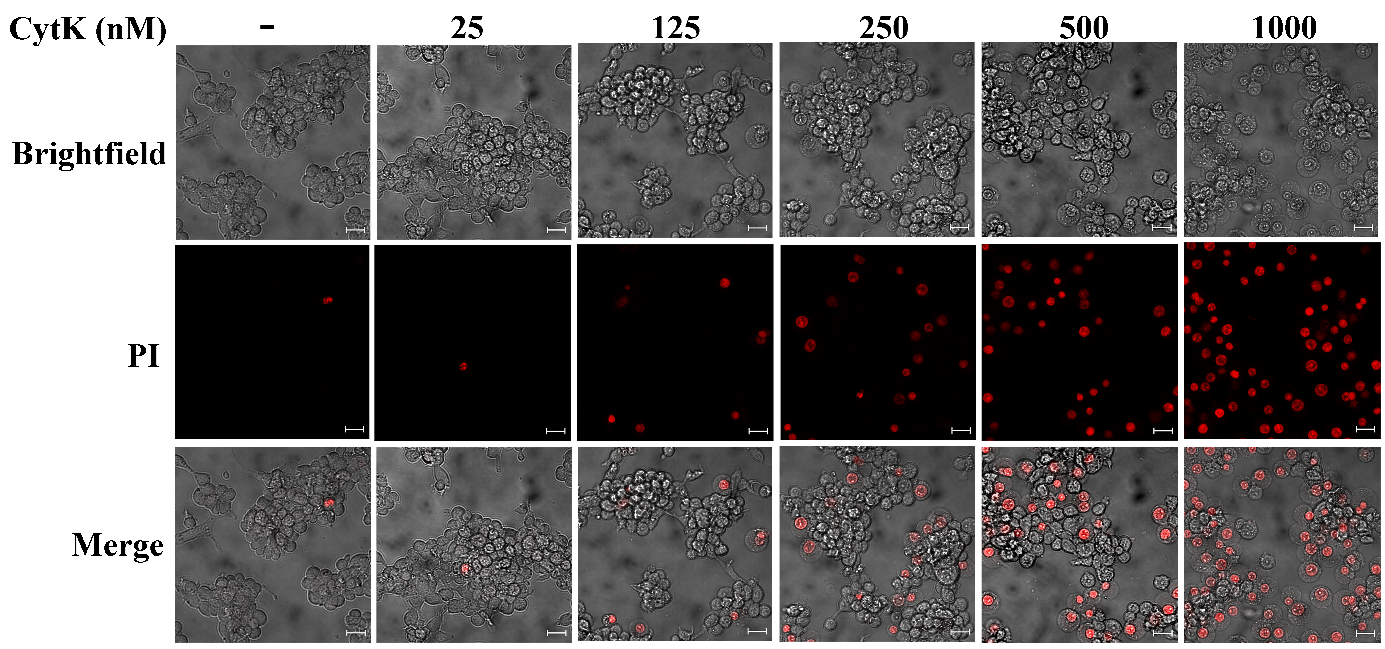
**

**Figure S3 Dose-dependent inhibition of CytK-induced cell death by zVAD and NSA**. PMA-differentiated THP-1 (dTHP-1) cells were pretreated with or without (-) increasing concentrations of the pan caspase inhibitor zVAD (A) or the GSDMD inhibitor necrosulfonamide (NSA) (B). The cells were then treated with CytK, and cell death was determined by measuring lactate dehydrogenase (LDH) release. Data are the means of triplicate experiments and shown as means ± SD. **p<0.01; ***p<0.001; ****p<0.0001; one-way ANOVA with Dunnett’s multiple-comparisons test. n.s, no significance.


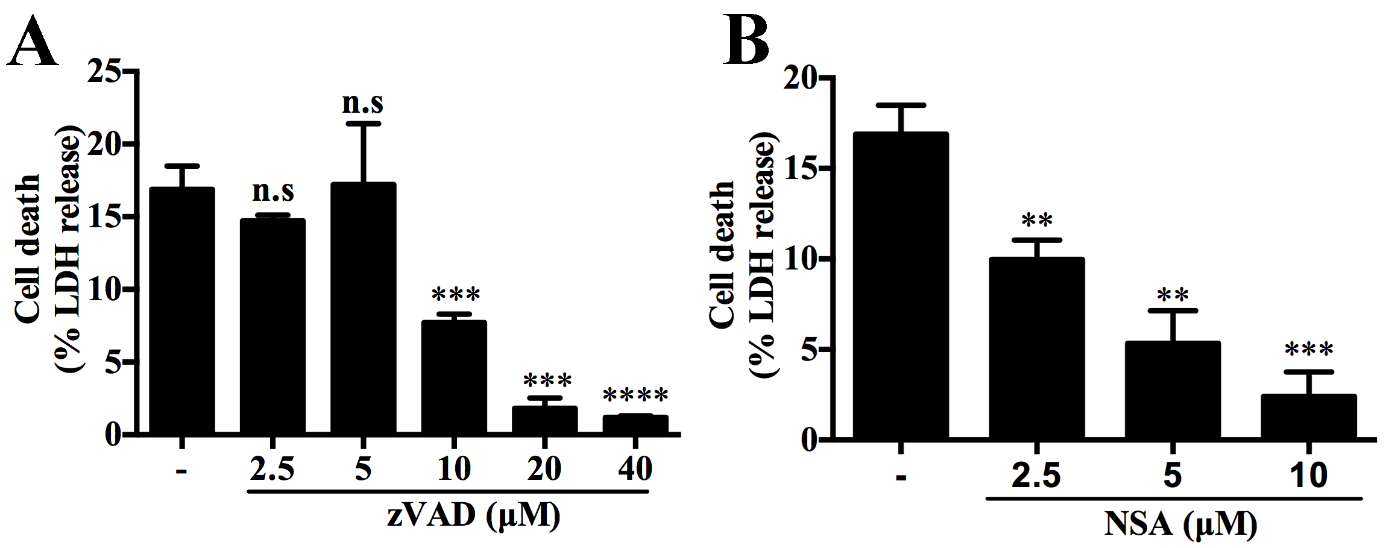


**Movie S1** The time-lapse images of PMA-differentiated THP-1 (dTHP-1) cells treated with CytK. Time: hh:mm:ss.
