# Supplementary figures and images for: Bacillus cereus cytotoxin K triggers gasdermin D-dependent pyroptosis
Source: Cell Death Discov. 2022 Jul 4;8:305. doi: 10.1038/s41420-022-01091-5 (PMC9253000; doi:10.1038/s41420-022-01091-5)

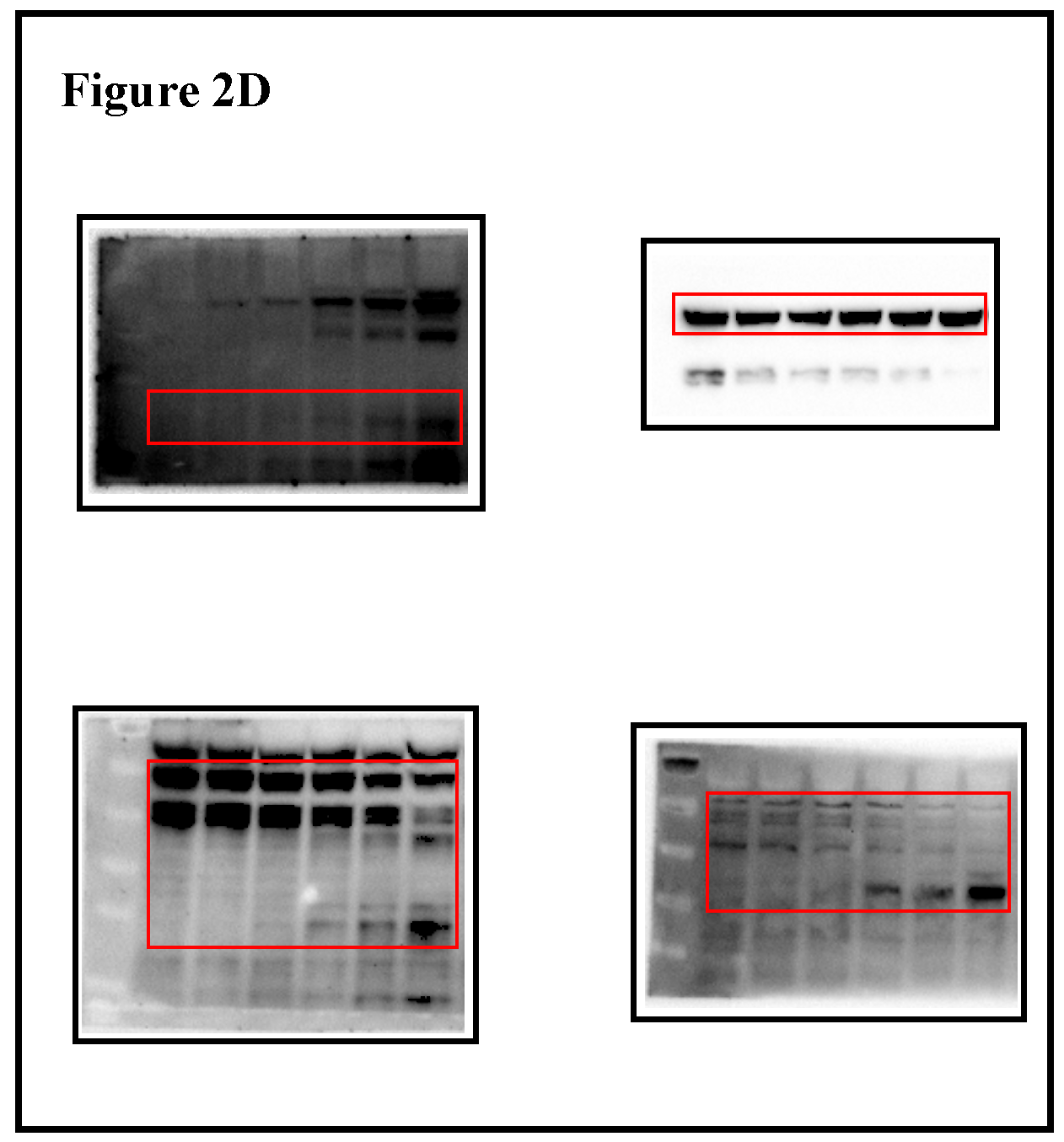


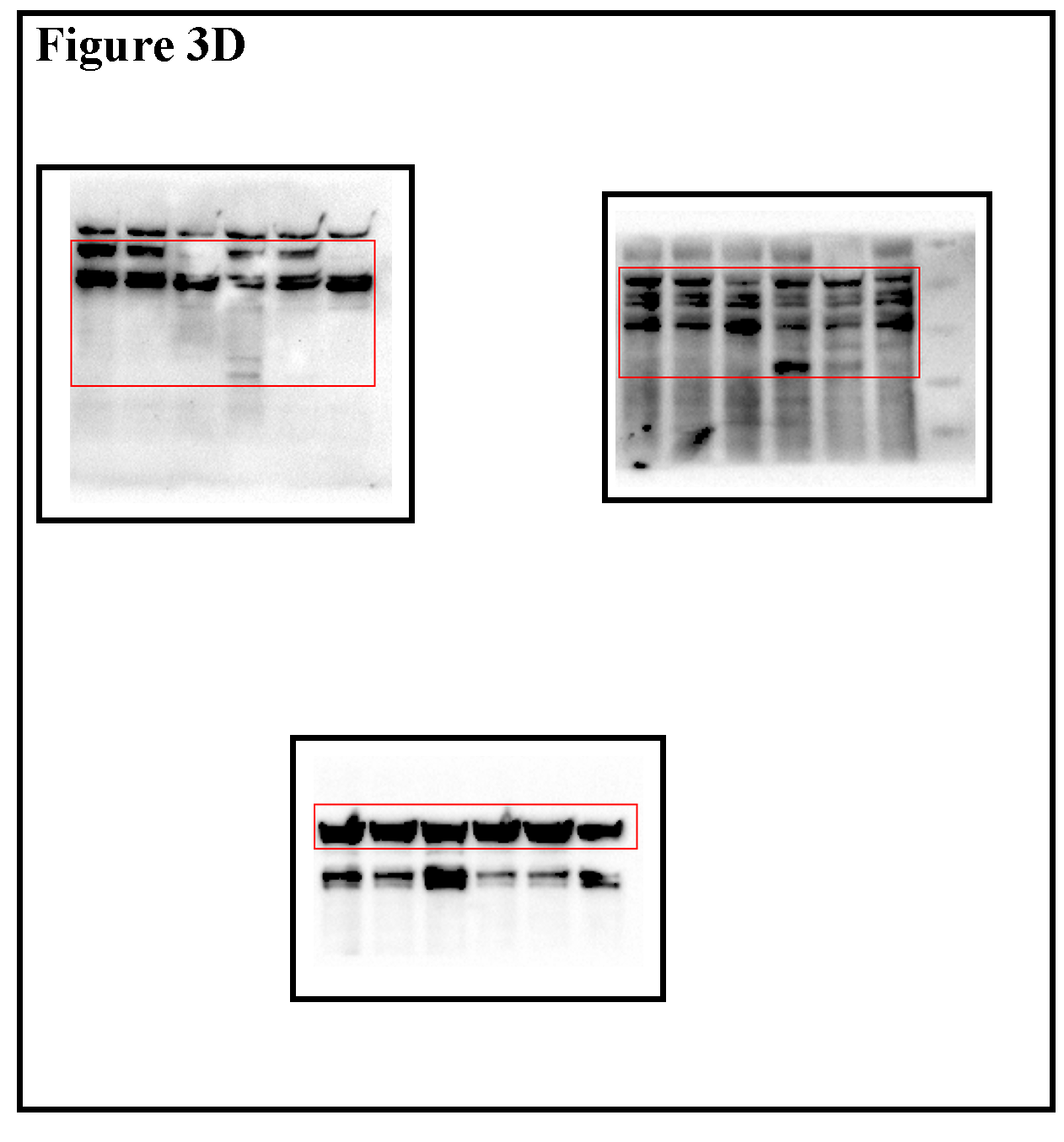


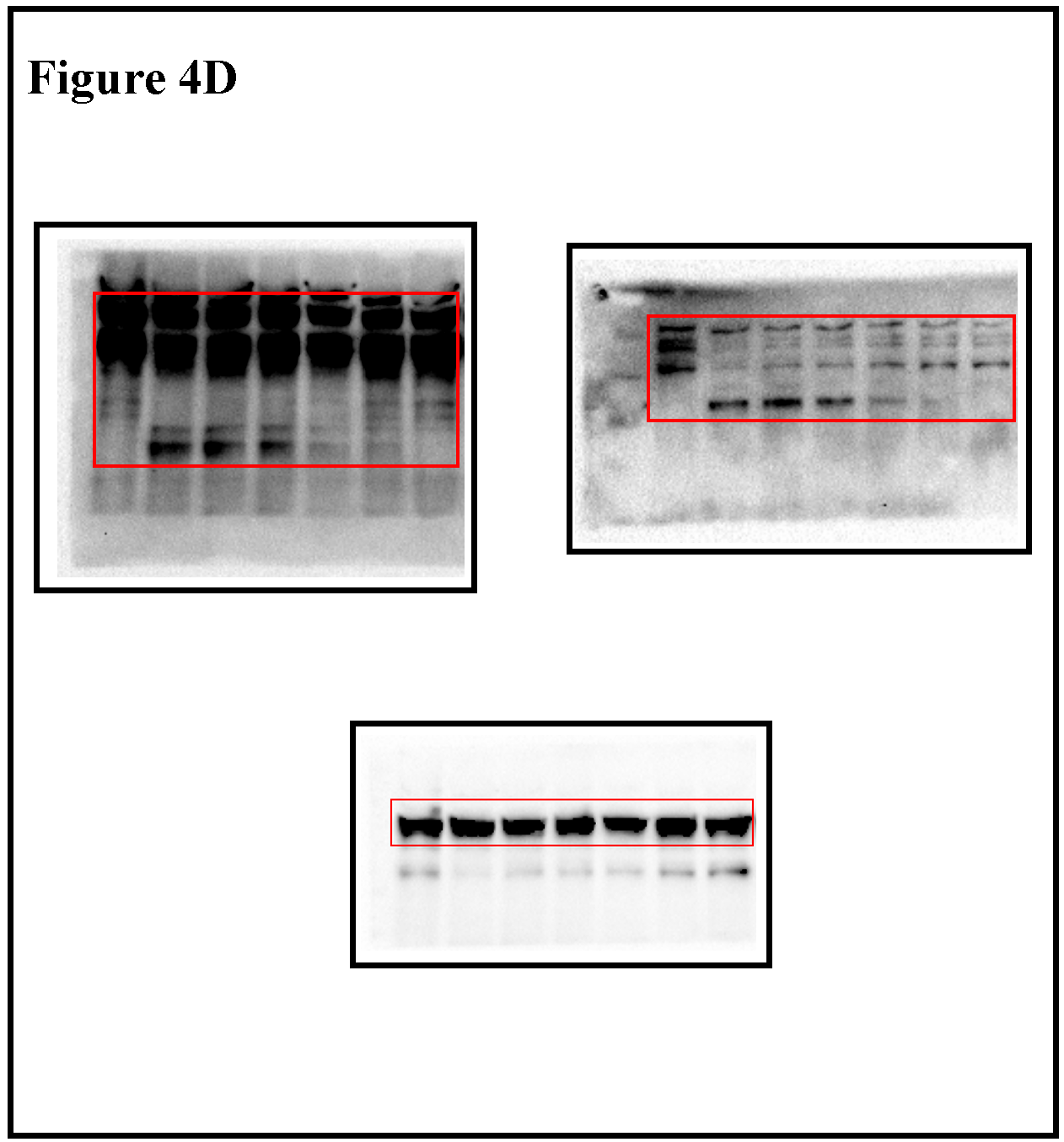


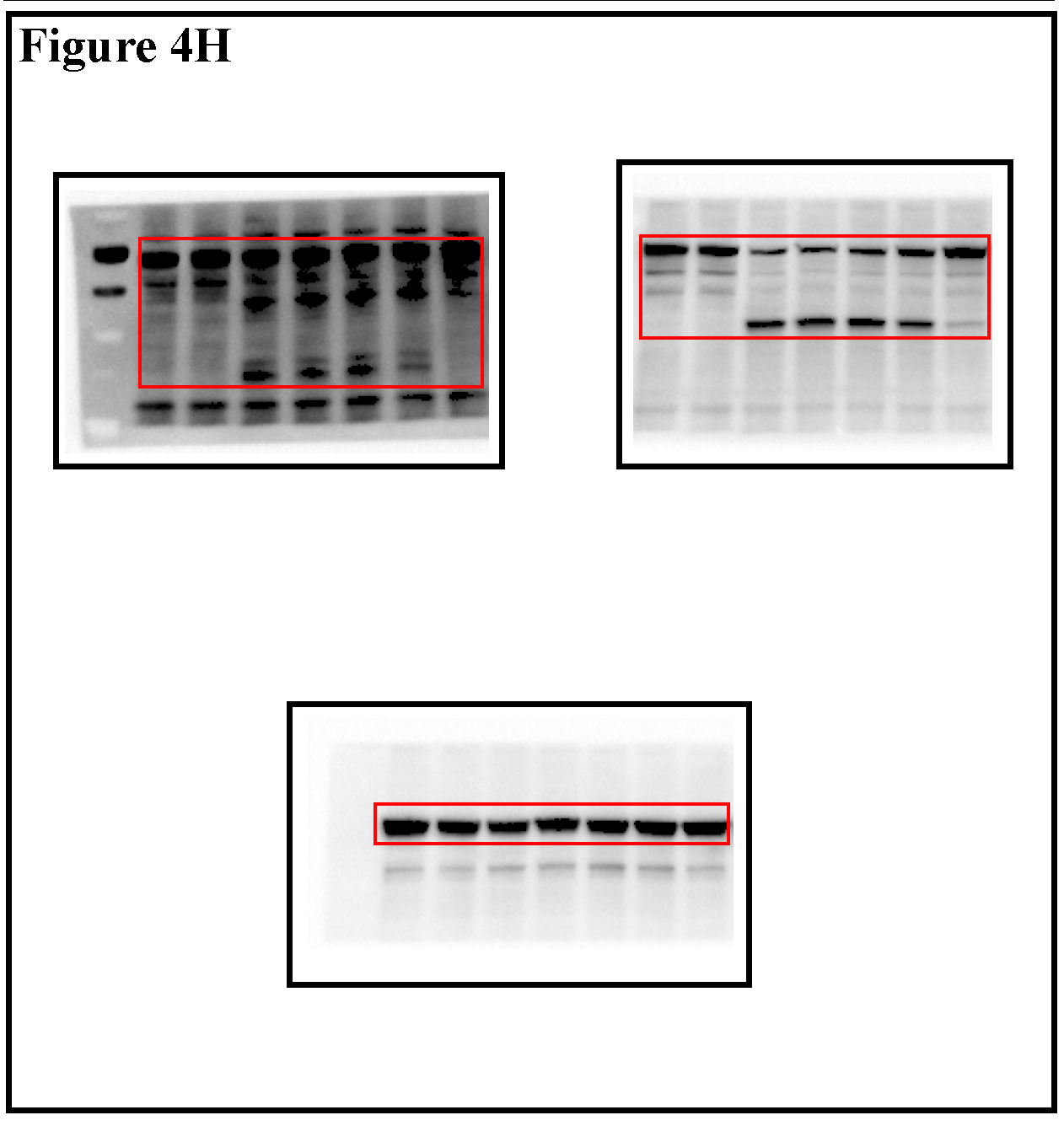


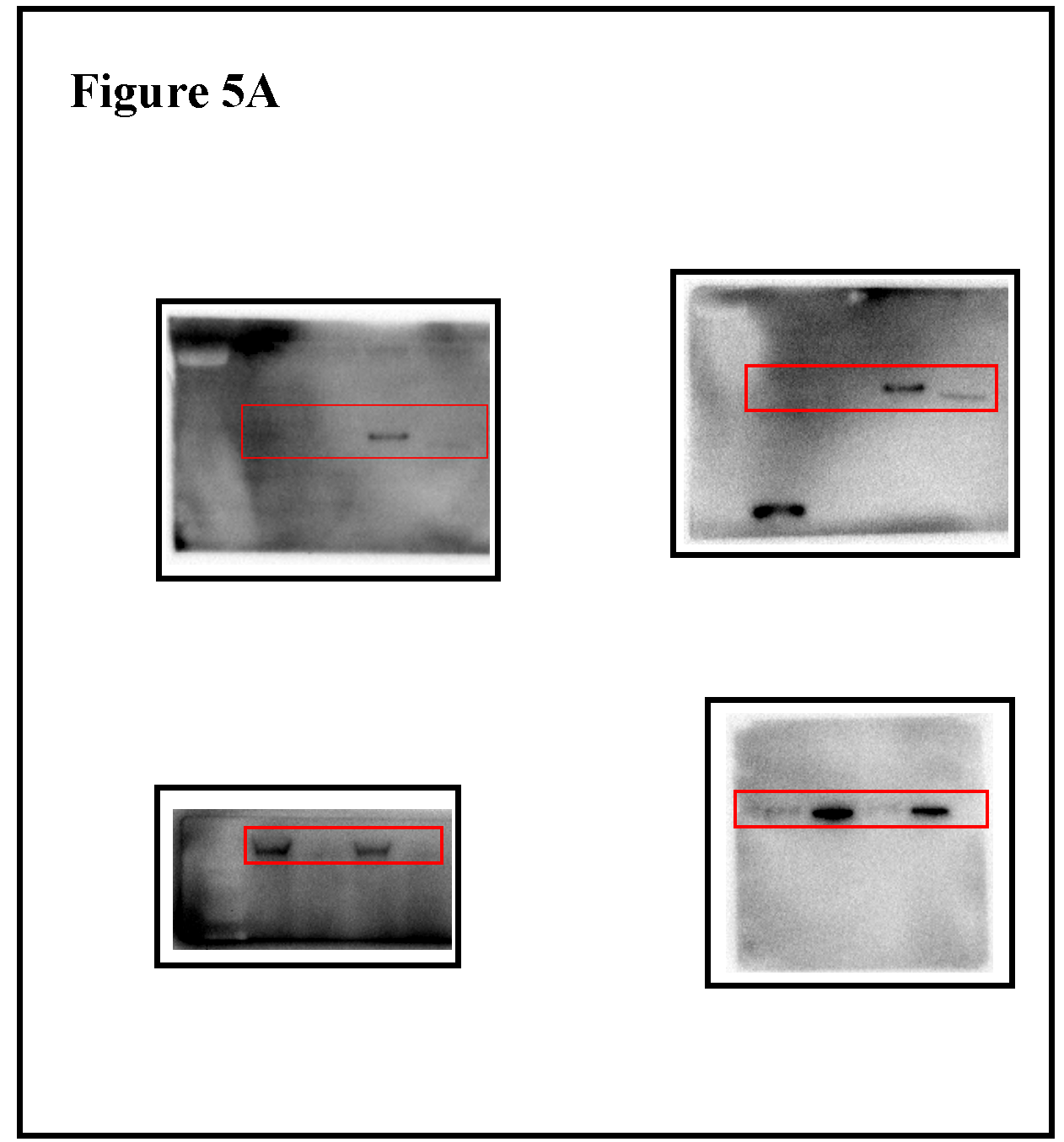


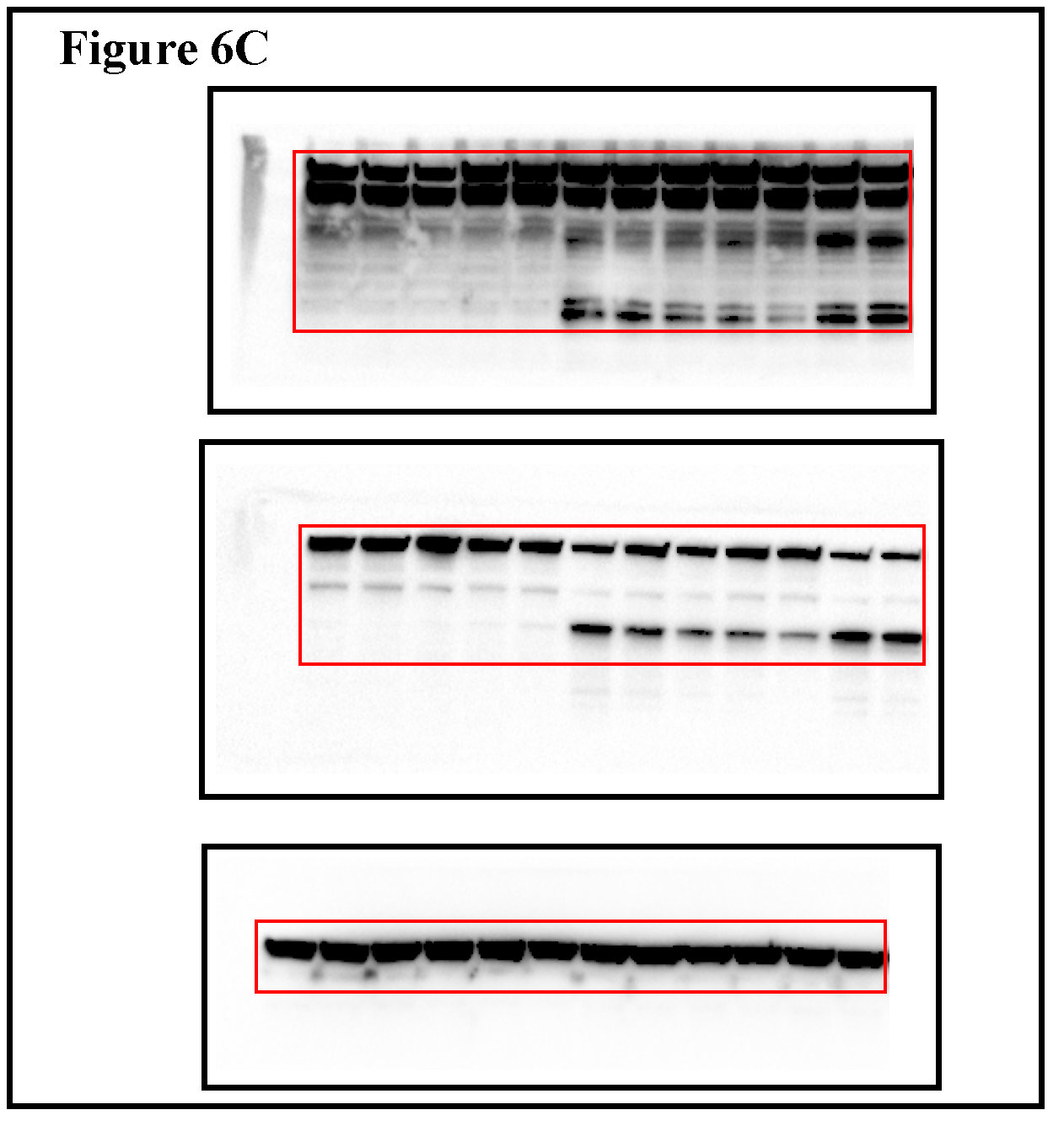

Supplement: Supplementary file 3 — Original Data File [file 41420_2022_1091_MOESM3_ESM.docx]
